# Deep-learning based 3D birefringence image generation using 2D multi-view holographic images

**DOI:** 10.1038/s41598-024-60023-8

**Published:** 2024-04-30

**Authors:** Hakdong Kim, Taeheul Jun, Hyoung Lee, Byung Gyu Chae, MinSung Yoon, Cheongwon Kim

**Affiliations:** 1 Department of Digital Contents, Sejong University, Seoul, Korea; 2 Department of Software Convergence, Sejong University, Seoul, Korea; 3 Communication and Media Research Lab, ETRI, Daejeon, Korea; 4 Department of Software, Sejong University, Seoul, Korea

**Keywords:** Applied optics, Optical techniques

## Abstract

Refractive index stands as an inherent characteristic of a material, allowing non-invasive exploration of the three-dimensional (3D) interior of the material. Certain materials with different refractive indices produce a birefringence phenomenon in which incident light is split into two polarization components when it passes through the materials. Representative birefringent materials appear in calcite crystals, liquid crystals (LCs), biological tissues, silk fibers, polymer films, etc. If the internal 3D shape of these materials can be visually expressed through a non-invasive method, it can greatly contribute to the semiconductor, display industry, optical components and devices, and biomedical diagnosis. This paper introduces a novel approach employing deep learning to generate 3D birefringence images using multi-viewed holographic interference images. First, we acquired a set of multi-viewed holographic interference pattern images and a 3D volume image of birefringence directly from a polarizing DTT (dielectric tensor tomography)-based microscope system about each LC droplet sample. The proposed model was trained to generate the 3D volume images of birefringence using the two-dimensional (2D) interference pattern image set. Performance evaluations were conducted against the ground truth images obtained directly from the DTT microscopy. Visualization techniques were applied to describe the refractive index distribution in the generated 3D images of birefringence. The results show the proposed method’s efficiency in generating the 3D refractive index distribution from multi-viewed holographic interference images, presenting a novel data-driven alternative to traditional methods from the DTT devices.

## Introduction

The 3D holographic microscope, also known as Optical Diffraction Tomography (ODT), makes significant contributions to cell observation by visualizing internal cell structures without the need for staining, unlike conventional techniques such as bright-field and electron microscopy. The traditional staining process not only consumes time and effort but can also cause damage or deformation to cells. ODT operates by measuring the refractive index of the sample, allowing imaging without causing harmful effects on the sample.

Recently, a dielectric tensor tomography (DTT) technique that can visualize birefringence properties was proposed^1,2^. DTT further has developed imaging technology by expressing both the internal shape of the sample and birefringence information according to the polarization direction. DTT involves the conversion of 2D holographic interference pattern images into 3D birefringence images through a combination of microscope hardware and formula-based programs.

In this study, a deep learning model is proposed for the generation of 3D birefringence images. The method begins with acquiring a set of multi-viewed 2D holographic interference pattern images along with ground truth 3D birefringence images using the DTT microscopy observing LC droplets. Subsequently, the designed model is trained using this data. The performance of the trained model is evaluated by comparing the ground truth (3D birefringence images) from the microscope with the estimation result from the proposed model. This evaluation includes both evaluating the quantitative and qualitative results. Finally, the study analyzes and discusses the results obtained from the experiments.

Our proposed method contributes in two main aspects when compared to existing formula-based methods. Firstly, it represents the initial attempt to generate 3D birefringence using a deep learning-based approach with 2D multi-view holographic images. Secondly, the time needed to generate birefringence is significantly reduced compared to existing formula-based methods.

## Related work

### 3D image processing

Generative models are being used in the field of 3D image processing^3^. A study proposes the use of a model known as 3DGAN to generate 3D objects^4^, and another study extends this 3DGAN to propose a method for more detailed 3D object reconstruction^5^. To perform inpainting on damaged 3D objects, a study has been proposed that combines Generative Adversarial Network (GAN) and Recurrent Neural Network (RNN) models^6^. Additionally, there is research that presents a GAN model for restoring full 3D objects using a single depth view where some parts of the 3D object are damaged^7^. In an attempt to enhance the restoration quality, methods employing new activation functions and rendering techniques have been explored^8^.

GANs are not limited to image generation; they are also employed for object detection^9^ or segmentation^10^ of existing images, contrary to their original purpose. These applications, particularly in the context of autonomous driving, are crucial technologies. For example, there are studies^11,12^ that detect cars, pedestrians, and other objects in images using depth maps representing the distance of objects from the camera. Additionally, to address the disparity between image-based object detection and LiDAR-based object detection, there are studies^13–16^ that assume the importance of the representation of 3D scenes. This research focuses on methods for transforming between these two representation approaches and exploring the relationship between 2D image coordinates and 3D point clouds.

Huang et al.^17^ introduced a 3D-CNN designed for the segmentation of voxels within 3D images. This method offers the advantage of efficiently handling substantial data volumes. Subsequently, a novel approach emerged, merging reinforcement learning and RNN techniques with the 3D-CNN, enabling object localization, segmentation, and classification^18^. Moreover, another technique was proposed to represent 3D space at a high resolution, ensuring efficient memory utilization while preserving resolution integrity^19^. What unites these prior studies and the proposed method is their utilization of deep learning methodologies to process 3D images. However, while the aforementioned studies use a standard RGB color system, compatible with general-purpose image deep learning models, this study uses birefringence images that do not conform to an RGB color system. As a result, these images necessitate a preprocessing step to be incorporated into the model.

### Holographic tomogram

Cryo-electron microscopy (Cryo-EM) analyzes the three-dimensional structure of biological samples at low temperatures by freezing them, leveraging the principles of ice formation^20^. X-ray crystallography utilizes X-ray diffraction to determine the three-dimensional structure of proteins or biological molecules^21^.

Lee et al.^22^ introduced a non-invasive method for diagnosing pathology by observing living cell structures. This phase microscopy technique employs 2D digital holography technology, necessitating constant internal sample conditions. To overcome this limitation, X-ray Computer Tomography (X-Ray CT) was proposed^23–25^. The Helmholtz equation used in X-ray CT represents the wave equation for a single wavelength, allowing its principles to extend to light sources like laser light, akin to visible light^26^. Holographic tomographic microscopy operates with visible-range laser light^27^. While X-ray CT primarily gauges the X-ray absorption distribution of the target, holographic tomographic microscopy measures the target’s refractive index.

Joo et al.^28^ presented a method to extract quantitative phase information from spectral interference signals obtained in Optical Coherence Tomography (OCT) setups. They laid the groundwork for converting interference patterns into quantitative phase data and understanding the correlation between interference patterns and refractive index distribution.

Various methods have been proposed using RNN^29–31^ to represent cellular changes over time and techniques employing 3D holographic tomographic microscopy to reconstruct label-free images from 3D refractive index images^32,33^. These studies align with this research by focusing on restoring refractive index images. However, earlier studies captured interference patterns using holographic tomographic devices and generated refractive index images through formula-based specialized programs^1,2^. In contrast, this study employs a data-driven approach using a deep learning model, taking interference patterns as input and generating refractive index images as output.

## Results

**Figure 1:**
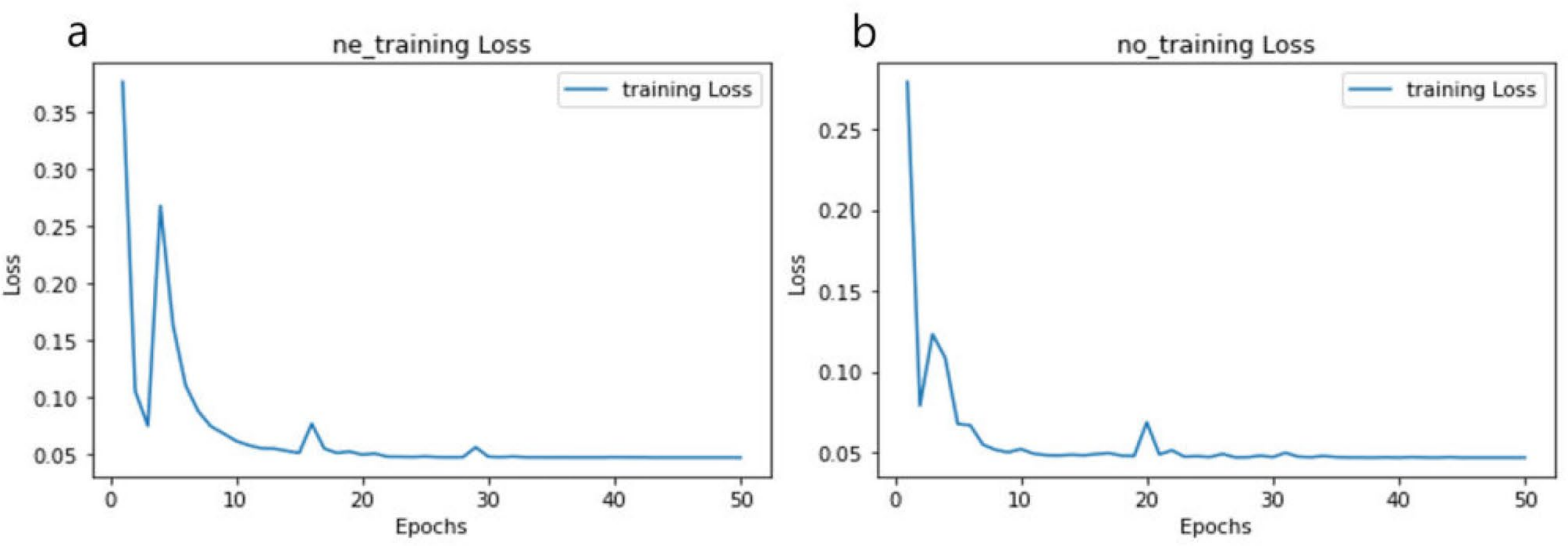
Trend of the training loss MSE for (**a**) extraordinary ray and (**b**) ordinary ray.

The input data we used for the proposed models’ training, with a size of $512 \times 512$, and the output data, with a size of $332\times 332 \times 80$, is considered significantly large for standard model’s training dataset. We performed machine learning tasks using the ASUS ESC8000-G4 series equipped with Nvidia Titan RTX $\times$ 8. Following the learning process, we proceeded with quantitative and qualitative comparison experiments using reference data. Figure 1 illustrates the trend of loss values throughout the training phase.

We observed the loss trend during 50 epochs and confirmed that both extraordinary ray (ne) and ordinary ray (no) converged to an Mean Squared Error (MSE) of approximately 0.045. The loss consistently decreased until the 45th epoch, but after the 50th epoch, the loss stabilized around the same value. As there’s no established benchmark for birefringent volume generation, conducting precise quantitative comparisons with other studies remains limited. We compared it with the Peak Signal-to-noise ratio (*PSNR*)^34^ of Kim et al.^35^, a study that generated a single refractive index volume, and the results are presented in Table 1. The *PSNR* is defined as

1 $$\begin{aligned} PSNR = 10log\frac{s^{2}}{MSE} \end{aligned}$$

which is defined via the *MSE* and *s* (maximal signal value of a target), equal to 255 for the RGB color system. We calculated the *PSNR* after normalizing the model’s output which is real values refractive index to 0 255. Furthermore, we calculated the Structural Similarity Index (*SSIM*)^36^ to assess the structural similarity between the ground truth and the results generated by the model. *SSIM* is defined as

2 $$\begin{aligned} SSIM = \frac{(2\mu _{y}\mu _{y'}+c_{1})(2\sigma _{yy'}+c_{2})}{(2\mu _{y}^2+\mu _{y'}^2+c_{1})(\sigma _{y}^2+\sigma _{y'}^2+c_{2})} \end{aligned}$$

where $\mu _{y}$ as the average of ground truth(y); $\mu _{y'}$ the average of the estimated depth map(y’); $\sigma _{y}^2$ the variance of the ground truth(y); $\sigma _{y'}^2$ the variance of the estimated depth map(y’); $\sigma _{yy'}$ the covariance of ground truth(y) and estimated depth map(y’); and $c_{1}$, $c_{2}$ are two variables for stabilizing the division with a weak denominator, which is defined as

3 $$\begin{aligned} c_{1} = (k_{1}L)^{2}, c_{2} = (k_{2}L)^{2} \end{aligned}$$

where *L* is the dynamic range of the voxel values (normally $$2^{bit per voxel}$$-1); and $$K_{1}$$ and $$K_{2}$$ are defined as default values of 0.01 and 0.03, respectively.

**Table 1:** Quantitative comparison of the proposed model and single refractive index volume generation model (higher values for both metrics indicate better performance.).

	Direction of polarization	PSNR (dB)	SSIM
Proposed method	Extraordinary ray	28.07	0.9108
	Ordinary ray	28.31	0.9186
Kim el al.^35^	Single ray	10.17	$\times$

**Figure 2:**
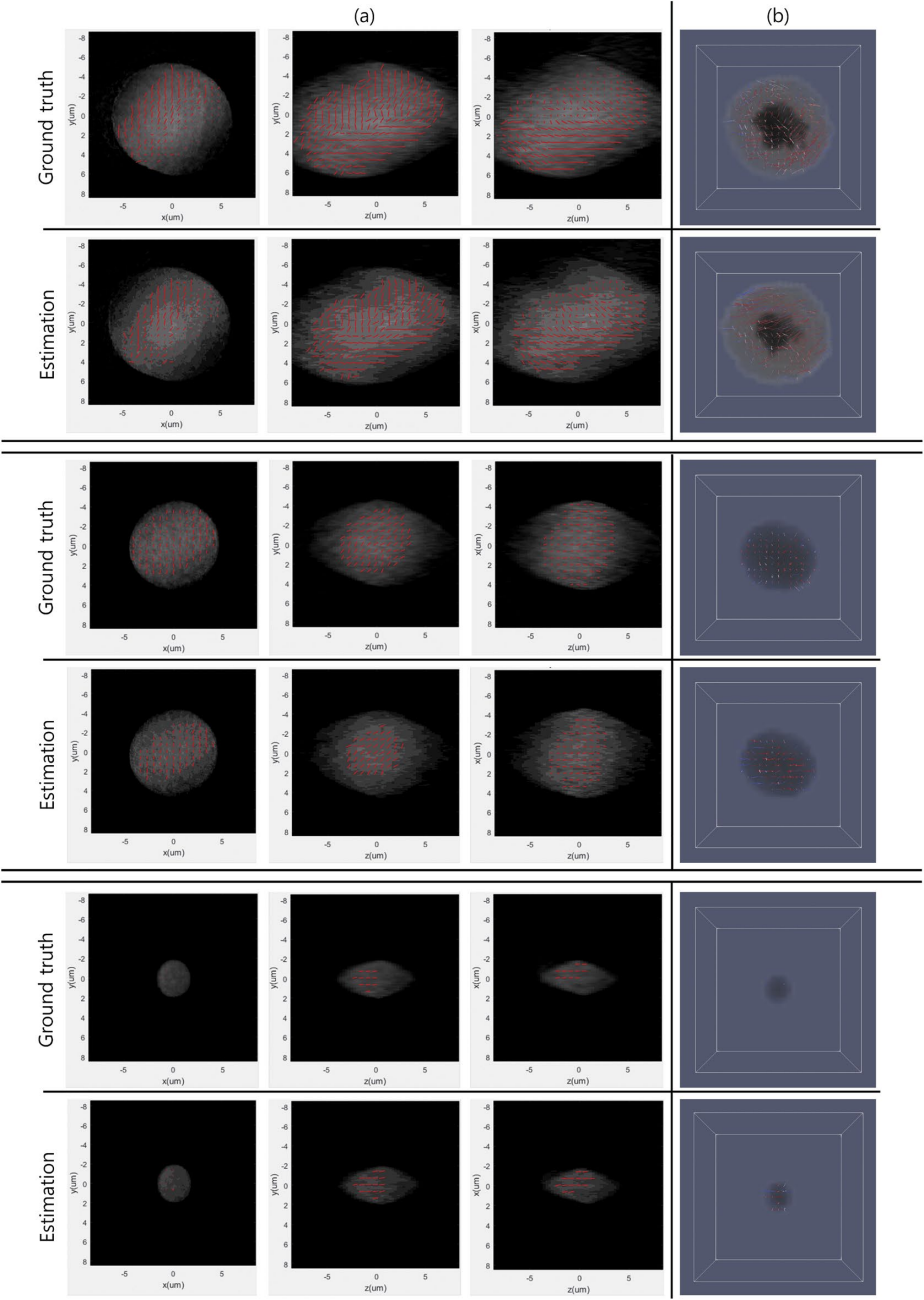
Visualization result for qualitative comparison between proposed method’s results and ground truth. (**a**) Liquid crystal droplet with representation of internal directional information according to *xy, zy, zx* slices. red lines indicate the direction of each particle. (**b**) 3D visualization for Liquid crystal droplet with each particle’s directional information. The 360-degree rotation for (**b**) can be found as Supplementary Videos  S1–S6 online..

We confirmed that the proposed method achieved a higher *PSNR* despite handling a larger volume resolution than Kim et al.^35^’s method, and the proposed method generated a birefringence volume, whereas Kim et al.^35^’s method generated a single refractive index volume. *SSIM* was also measured as a satisfactory level. Of course, it cannot be certain because there are no benchmark papers using a deep learning-based method to generate a birefringence volume and measure *SSIM*, but at least the luminance, contrast, and structure between the ground truth and model results are approximately 91% similar through the measured *SSIM*. Additionally, we qualitatively compared the results of the proposed model with the ground truth to confirm that the proposed method actually generates the birefringence volume well, and the results are shown in Fig. 2.

To show that the proposed deep learning-based method is superior to the existing formula-based method^1,2^ in terms of time, we conducted a birefringence generation experiment using both methods on the same sample in the same environment (ASUS ESC8000-G4 series with 4 Nvidia’s Titan RTX). Table 2 shows the experiment result. The generation of 3D birefringence from 2D multi-view holograms using the formula-based method^1,2^ takes approximately 35 minutes and 15 seconds, while the proposed method accomplishes the same task in approximately 5 minutes and 10 seconds. We conducted measurements on 10 samples, repeating each measurement 5 times using both the formula-based method^1,2^ and the proposed method, and then calculated the average after subtracting the maximum and minimum values.

**Table 2:** Comparison of time to generating 3D birefringence.

	3D birefringence generation	Visualization
Formula-based^1,2^ (min’ s”)	35’ 15”	3’ 28”
Deep learning-based (min’ s”) (proposed method)	5’ 10”	3’ 28”

## Discussion and conclusions

We acquired training data consisting of 2D interference pattern images and 3D birefringence images using a holographic microscope and then trained a proposed model that uses the generated training data as input and output data. Through the proposed model, we visualized the model’s output that represents the internal view of the target and the direction information of particles of the target. The method begins with acquiring a set of multi-view 2D interference pattern images together with 3D birefringence images using a holographic microscope. To efficiently train the proposed model, 3D birefringence images obtained through a microscope system were quantified. Afterward, we trained the proposed model until 50 epochs, when the loss was no longer reduced. The generation results of the ground truth and the proposed model were compared quantitatively and qualitatively. Through comparison with another previous study^35^ that estimated a single refractive index, we confirmed that the proposed model achieved a higher *PSNR* and that luminance, contrast, and structure were more than 91% similar to the ground truth. The visualization results for *xy, zy*, and *zx* slices and 3D visualization results were presented. These two visualization results include a representation of the direction of particles inside the liquid crystal.

The contributions of this study are as follows. First, the proposed method is the first attempt to generate 3D birefringence information from 2D hologram information through a data-based approach. Second, the time needed to generate birefringence is significantly reduced compared to existing formula-based methods. By acquiring data, training deep learning models, quantitative testing, and visualization, it contributes to the activation of datasets and benchmarks in the birefringence domain. Third, the proposed data-driven method is useful as a replacement or supplement to the formula-driven DTT methods by quickly generating the birefringence volume of the object from the 2D interference pattern images.

The limitations of the proposed method are the background noise and slight distortion near the border between the object and the background. background noise can be solved by adding a denoising block. To overcome distortion near the border between the object and the background, it is expected that an approach increases the difference between the background and the object by performing normalization on the layers within the model. In addition to solving the above two limitations, we are planning future research on a super-resolution to increase the resolution of the 3D birefringence volume. High-resolution 3D birefringence imaging technology can visualize the internal 3D shape of materials such as liquid crystals, biological tissues, and silk fibers in high quality in a non-invasive manner. It is expected to be useful in the semiconductor, display industry, optical component devices, and biomedical diagnosis. Our intended scope for this study was to utilize liquid crystal droplets. However, in a follow-up study, we plan to extend the current proposed model to tasks that involve reconstructing both the internal and external shapes of blood cells.

## Methods

We utilized a formula-based approach^1,2^ solely to generate the ground truth for model training. Subsequently, the training and testing of the proposed model were performed using a deep learning-based method, which is a data-driven approach.

### Data acquisition

**Figure 3:**
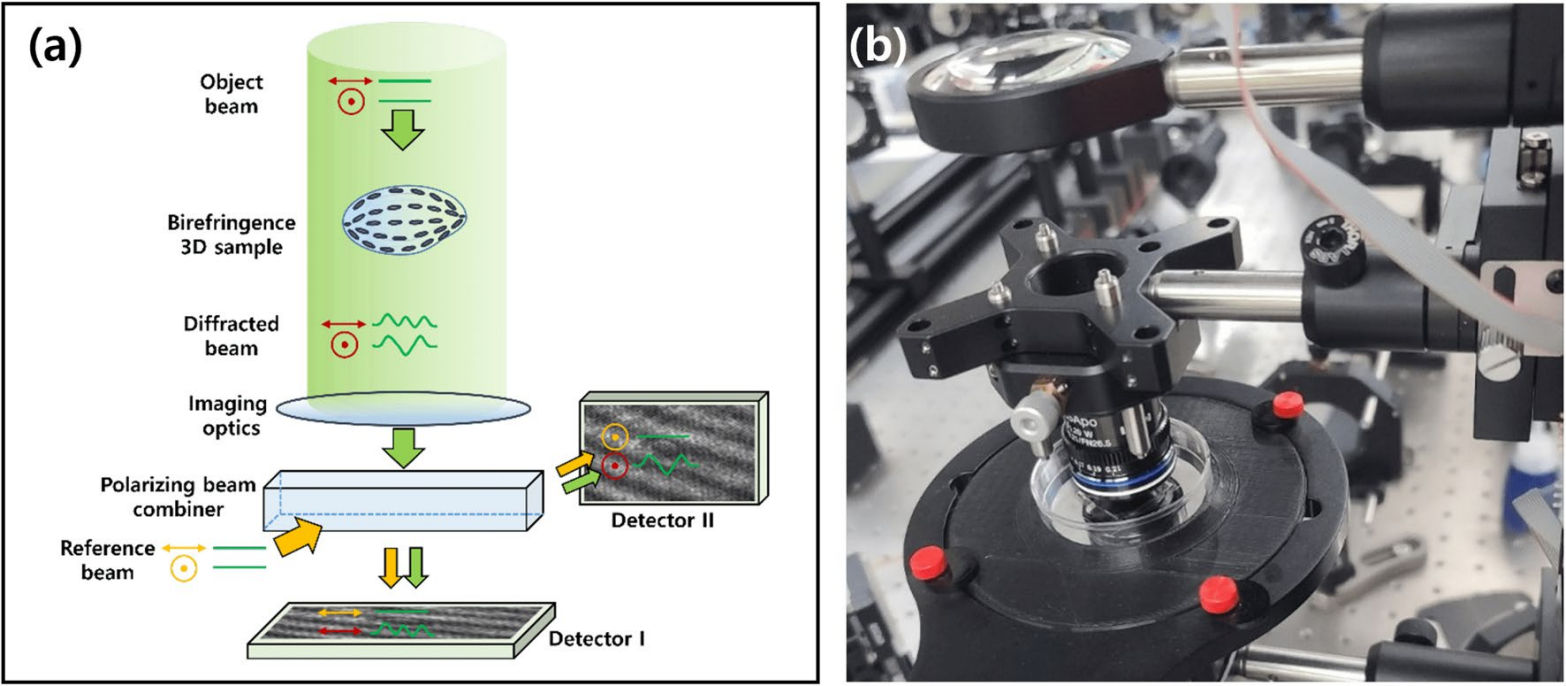
(**a**) A schematic of the DTT measurement microscope where the object beam diffracted from a birefringence 3D sample is measured using a polarizing-sensitive interferometer. (**b**) The microscope used for dataset acquisition (2D-interference pattern images and 3D-birefringence image).

To acquire training data for the birefringence images, interference patterns need to be captured from two different polarization directions. Figure 3 shows a holographic tomogram device that we directly constructed for capturing interference patterns. For each sample, 37 images were captured in the extraordinary ray polarization direction and 37 images in the ordinary ray polarization direction, resulting in a total of 74 interference pattern images. This process was applied to a total of 750 samples. The 3D birefringence images were obtained using formula-based microscopy-specific software^1,2^ after acquiring the interference pattern images.

A model for generating birefringence images differs from general image models because birefringence images follow the refractive index representation rather than the standard RGB representation. In this study, voxel values in birefringence images are constrained to a specific range of real numbers. When measuring the samples of liquid crystal (LC) droplets we used in the study, their 3D birefringence index values were generated within a specific range between 1.54 and 1.57 in the initial data acquisition process for the ground truth. This range was designated because it represents the optimal range for observing the samples^1,2^. Although the 3D birefringence image acquired from the microscope visually represents 3D birefringence information, without quantifying the values of each voxel, the model cannot be effectively trained. Therefore, we converted birefringence images into 3D numpy arrays to obtain quantitative values for each voxel, designing the model to learn from these numpy arrays. Consequently, for a total of 750 samples, we obtained a dataset comprising 74 interference patterns of size $512 \times 512$ per sample and 3D birefringence values of size $332 \times 332 \times 80$.

### Proposed model

**Figure 4:**
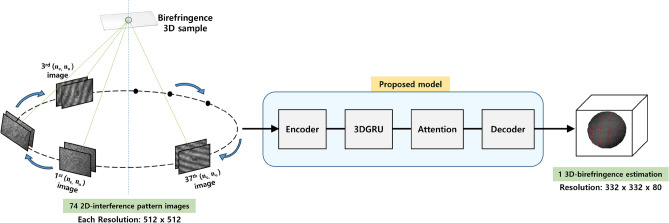
The process of generating 3D birefringence through the proposed model using 2D interference patterns.

The proposed model consists of four components, such as Encoder-3DGRU-Attention-Decoder, as shown in Fig. 4. Initially, before the input data enters the encoder, interference pattern images are grouped into sequences. Through mean operations, the information is condensed into the sequences. The number of images in a sequence is determined by the window length, sequence length, and the number of input images. Here, the window length represents the number of interference pattern images grouped in one sequence, and the sequence length is the total number of sequences. The final sequence length remains the same if the sequence length is even. However, if the original sequence length is odd, the final sequence length is sequence length-1.

**Figure 5:**
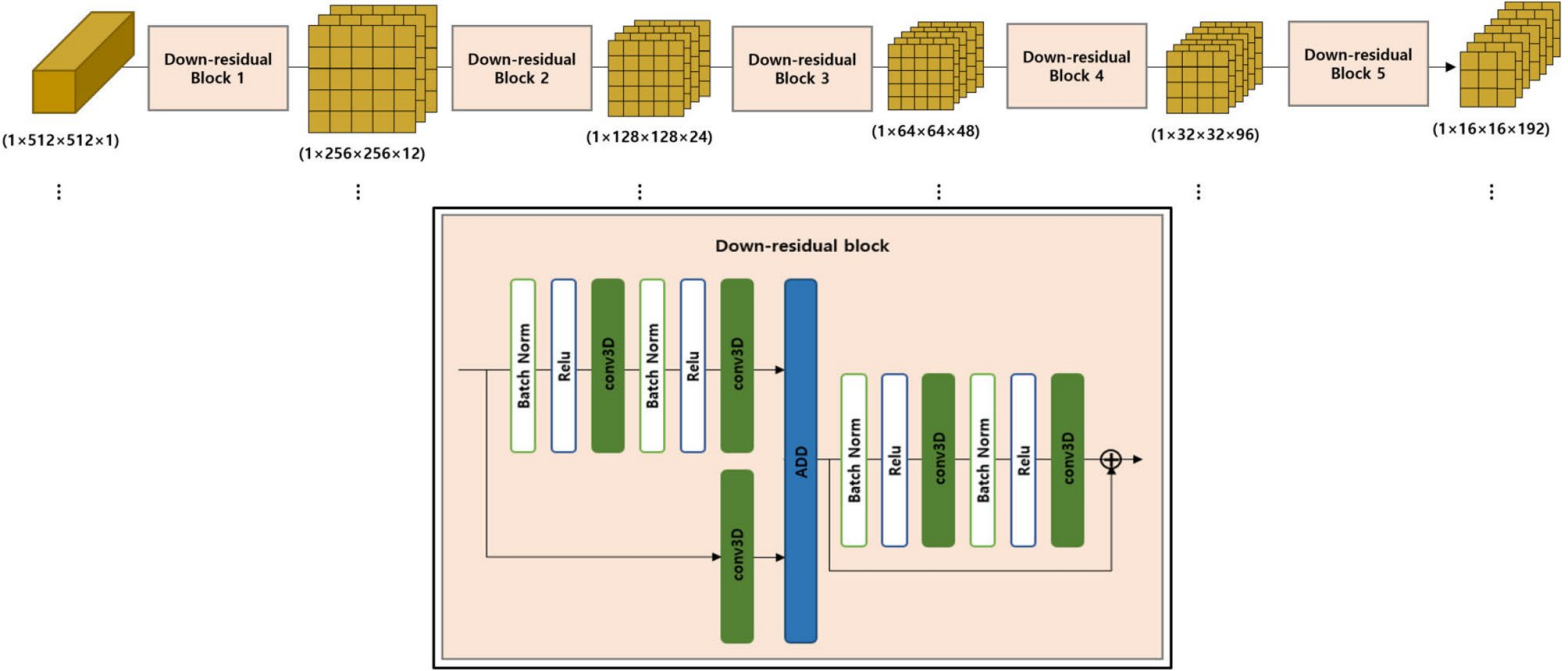
Encoder: Extract feature of input data using 5 down residual blocks.

As shown in Fig. 5, the encoder is consist of five down-residual blocks, sequentially outputting feature maps. The layers of the down-residual block consist of a batch normalization layer, ReLU activation, a 3D convolution layer, and an Add layer. In the Add layer within the block, the process involves adding the generated vector, which has passed through two sets of Batch normalization-ReLU-3D convolution layers, with the feature map generated through the Conv3D layer. This step ensures a smooth integration of the original information into the extracted information. As the block is sequentially passed through, the height and width of the feature maps decrease, while the channel count increases. Passing through the convolution layer within the block enables the learning of spatial features of beads sampled within the interference pattern, resulting in multiple feature maps. As a result, six feature maps of size $1\times 16\times 16\times 192$ are generated. The feature map produced by the encoder is then used as the input for the 3DGRU structure.

**Figure 6:**
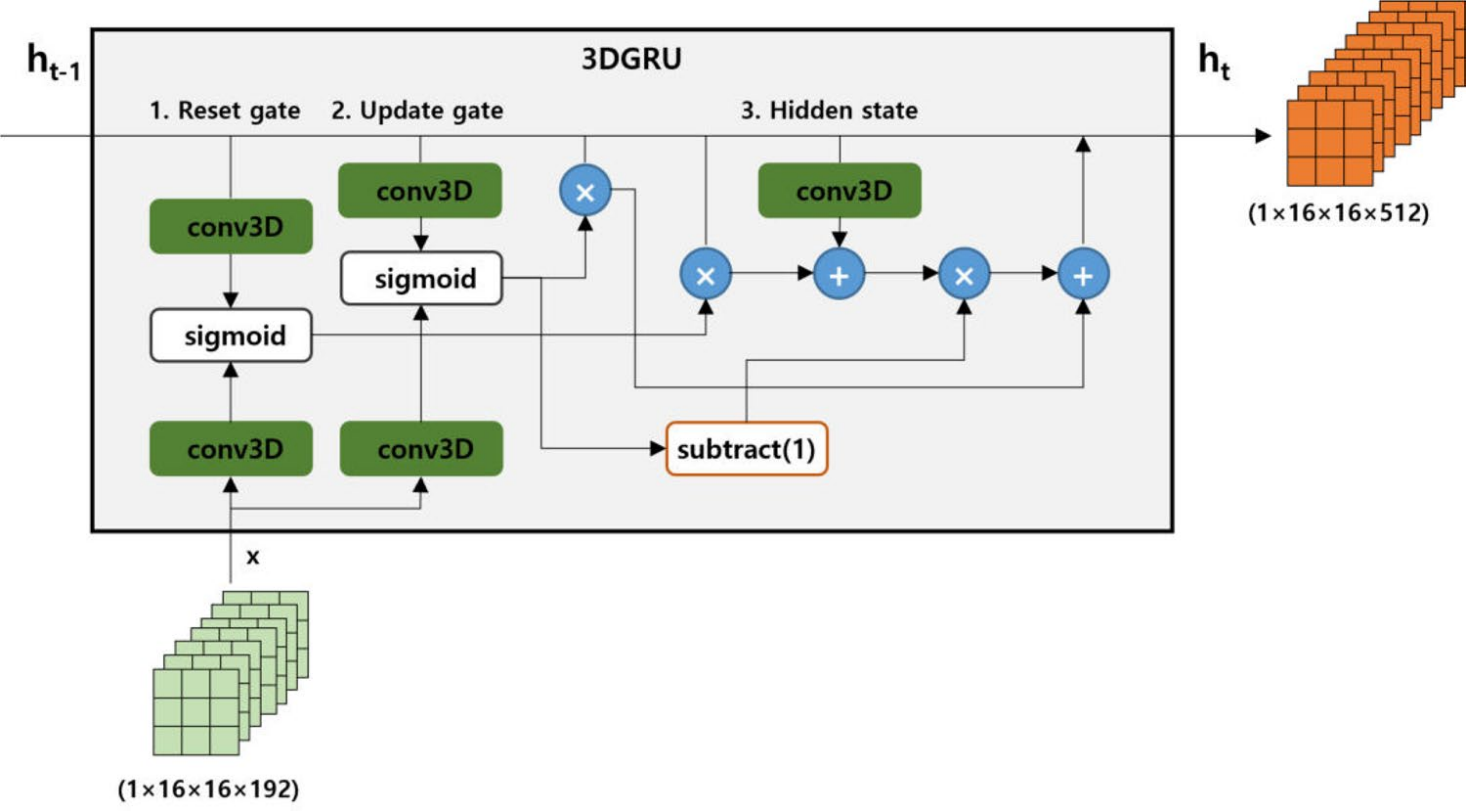
3DGRU (3D Gated Recurrent Unit): Learning spatial patterns through 3D convolution.

The 3DGRU^33^ presented in Fig. 6 is a model that considers spatial patterns for 3D sequences and accounts for temporal dynamics. Since we capture interference pattern images of liquid crystal bead samples at different time points with a constant time lag from a specific polarization direction, the 3DGRU is well-suited for processing the 3D sequence feature information. Therefore, we have included the 3DGRU in the proposed model to effectively handle the spatio-temporal characteristics of the liquid crystal bead samples. 3DGRU follows the structure of the general recurrent neural network Gated Recurrent Unit (GRU) and consists of Reset gate and Update gate. In the Reset gate, operations are performed based on the current input and information from the previous time step, controlling how much of the current information should be used through the sigmoid function. The Update gate controls how much information from the previous time step $h_{t-1}$ should be output. In the Hidden state, after passing through the two gate structures, it considers the output of the update gate and the initial information from time step $h_{t-1}$ to calculate and output a new feature map of size $1\times 16\times 16\times 512$ corresponding to the new $h_{t}$.

**Figure 7:**
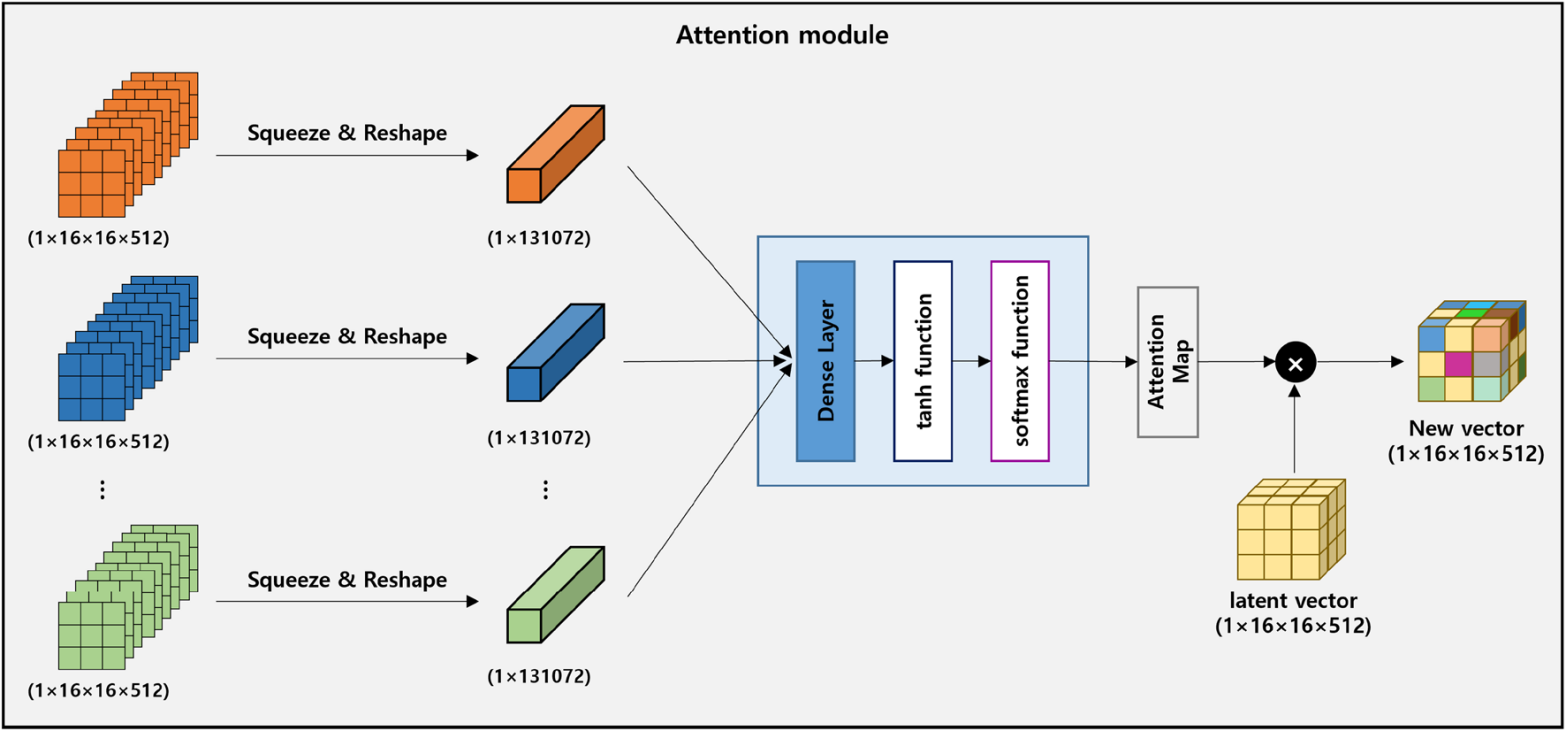
Attention: Learning locational feature information.

In Fig. 7, the attention module takes the feature maps obtained for each image, feeds them into the GRU, and adjusts their importance by considering the spatial features within the images. Weights are calculated for each position of the feature information that has gone through the GRU structure. This process allows the model to focus on specific locations. As a result, more attention is given to important areas, enabling the model to grasp the essential parts of the feature map. The feature map generated through 3DGRU is transformed into a set of $16 \times 16\times 51$2-sized feature maps, with the same number as the sequence length. Each $1\times 16\times 16\times 512$ feature map undergoes a reshaping process, being squeezed into a one-dimensional vector, which is then used as input for a dense layer. The vector output from the dense layer is used to produce an attention map through the application of Hyperbolic tangent and Softmax. The final vector is then generated through the multiplication operation between the attention map and the latent vector.

**Figure 8:**
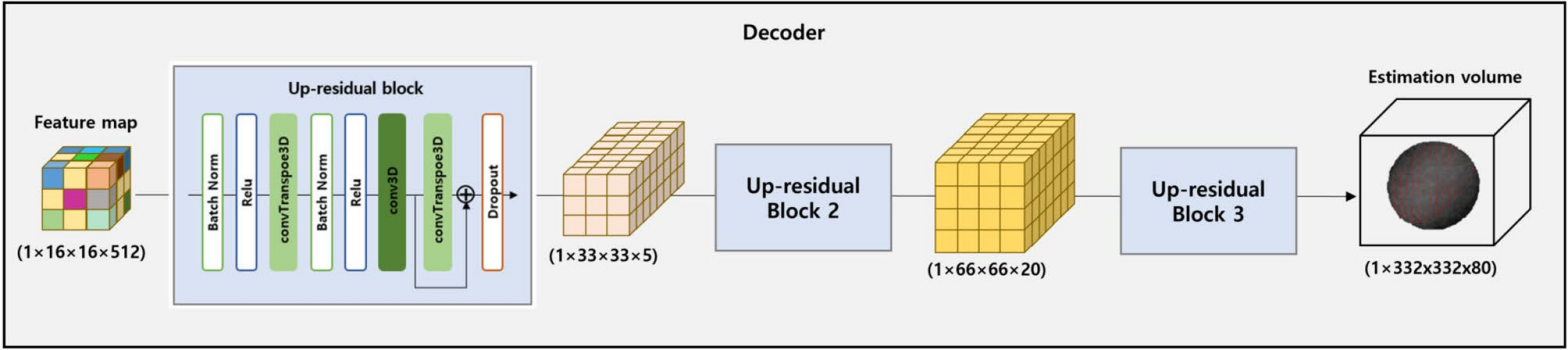
Decoder: Generate a 3D volume of ground truth size using 3 up residual blocks.

In Fig. 8, a 3D volume corresponding to the final output is generated from the feature maps produced by the preceding structures. Particularly, the model that pays attention to important areas through the Attention mechanism incorporates this information into the generated volume. This ensures that the generated volume accurately reflects the refractive index information for crucial regions in the input interference pattern images. In the decoder, multiple decoder blocks are sequentially applied to gradually increase the feature map size. The size of the feature map input to the decoder is $1\times 16\times 16\times 512$, where each dimension represents the layer, width, height, and channel. In the Up-residual block, the feature map passes through batch normalization and ReLU sequentially. Transpose convolution layers are then used to upsample the layer, width, and height dimensions of the feature map to a larger spatial size. Following this, the 3D convolution layer and skip connection structure help the output better represent information from the previous layer. Finally, the decoder generates a 3D refractive index volume of size $332\times 332\times 80$ through a total of 3 residual blocks. To ensure that the model’s training/test processes are under the same conditions as the data acquisition process, the output values at the final layer were constrained to the range of 1.54 to 1.57^37–39^. By designing the proposed model to have output values within the same constrained range during the data acquisition process, we discovered that the model maintained consistency, improved stability, and provided easier interpretation of the results.

### Learning parameters

During the training process of the 3D refractive index volume estimation model using 2D interference pattern images, Mean Squared Error (MSE) was employed as the loss function. MSE allows for an efficient comparison between the actual values and the model’s predictions for refractive indices at each coordinate. As the refractive index values for bead samples range between 1.54 and 1.57 as real numbers, MSE is suitable for quantitatively comparing the disparities between actual and predicted values. We utilized the Adam optimizer with a learning rate of 0.0001. Throughout the model training, a batch size of 2 was applied to the dataset, and the training was conducted for a total of 50 epochs based on the experimental conditions.

### Supplementary Information

Supplementary Legends.

Supplementary Video 1.

Supplementary Video 2.

Supplementary Video 3.

Supplementary Video 4.

Supplementary Video 5.

Supplementary Video 6.

## Data Availability

The datasets prepared for the current study are not publicly available since they are under license permitted only within the current study, but they could be available from the corresponding author upon reasonable request.

## References

[1] Shin S (2022). Tomographic measurement of dielectric tensors at optical frequency. Nat. Mater..

[2] Lee J, Shin S, Hugonnet H, Park Y (2022). Spatially multiplexed dielectric tensor tomography. Opt. Lett..

[3] Goodfellow, I. *et al.* Generative adversarial nets. *Adv. Neural Inf. Process. Syst.* **27** (2014).

[4] Wu, J., Zhang, C., Xue, T., Freeman, B. & Tenenbaum, J. Learning a probabilistic latent space of object shapes via 3d generative-adversarial modeling. *Adv. Neural Inf. Process. Syst.* **29** (2016).

[5] Smith, E. J. & Meger, D. Improved adversarial systems for 3d object generation and reconstruction. In *Conference on Robot Learning*, 87–96 (PMLR, 2017).

[6] Wang, W., Huang, Q., You, S., Yang, C. & Neumann, U. Shape inpainting using 3d generative adversarial network and recurrent convolutional networks. In *Proceedings of the IEEE international conference on computer vision*, 2298–2306 (2017).

[7] Yang, B. *et al.* 3d object reconstruction from a single depth view with adversarial learning. In *Proceedings of the IEEE international conference on computer vision workshops*, 679–688 (2017).

[8] Chan, E. R., Monteiro, M., Kellnhofer, P., Wu, J. & Wetzstein, G. pi-gan: Periodic implicit generative adversarial networks for 3d-aware image synthesis. In *Proceedings of the IEEE/CVF conference on computer vision and pattern recognition*, 5799–5809 (2021).

[9] Kim S-H, Hwang Y (2021). A survey on deep learning based methods and datasets for monocular 3d object detection. Electronics.

[10] He, Y. *et al.* Deep learning based 3d segmentation: A survey. arXiv preprint arXiv:2103.05423 (2021).

[11] Xu, B. & Chen, Z. Multi-level fusion based 3d object detection from monocular images. In *Proceedings of the IEEE conference on computer vision and pattern recognition*, 2345–2353 (2018).

[12] Ahmed, Z., Iniyavan, R. *et al.* Enhanced vulnerable pedestrian detection using deep learning. In *2019 International Conference on Communication and Signal Processing (ICCSP)*, 0971–0974 (IEEE, 2019).

[13] Wang, Y. *et al.* Pseudo-lidar from visual depth estimation: Bridging the gap in 3d object detection for autonomous driving. In *Proceedings of the IEEE/CVF Conference on Computer Vision and Pattern Recognition*, 8445–8453 (2019).

[14] Fu, H., Gong, M., Wang, C., Batmanghelich, K. & Tao, D. Deep ordinal regression network for monocular depth estimation. In *Proceedings of the IEEE conference on computer vision and pattern recognition*, 2002–2011 (2018). 10.1109/CVPR.2018.00214 PMC6607900 31274971

[15] Kim, Y. & Kum, D. Deep learning based vehicle position and orientation estimation via inverse perspective mapping image. In *2019 IEEE Intelligent Vehicles Symposium (IV)*, 317–323 (IEEE, 2019).

[16] Wang, H. *et al.* Normalized object coordinate space for category-level 6d object pose and size estimation. In *Proceedings of the IEEE/CVF Conference on Computer Vision and Pattern Recognition*, 2642–2651 (2019).

[17] Huang, J. & You, S. Point cloud labeling using 3d convolutional neural network. In *2016 23rd International Conference on Pattern Recognition (ICPR)*, 2670–2675 (IEEE, 2016).

[18] Liu, F. *et al.* 3dcnn-dqn-rnn: A deep reinforcement learning framework for semantic parsing of large-scale 3d point clouds. In *Proceedings of the IEEE international conference on computer vision*, 5678–5687 (2017).

[19] Riegler, G., Osman Ulusoy, A. & Geiger, A. Octnet: Learning deep 3d representations at high resolutions. In *Proceedings of the IEEE conference on computer vision and pattern recognition*, 3577–3586 (2017).

[20] Adrian M, Dubochet J, Lepault J, McDowall AW (1984). Cryo-electron microscopy of viruses. Nature.

[21] Smyth M, Martin J (2000). x ray crystallography. Mol. Pathol..

[22] Lee K (2013). Quantitative phase imaging techniques for the study of cell pathophysiology: From principles to applications. Sensors.

[23] Kim K (2016). Optical diffraction tomography techniques for the study of cell pathophysiology. J. Biomed. Photon. Eng..

[24] Kim T, Zhou R, Goddard LL, Popescu G (2016). Solving inverse scattering problems in biological samples by quantitative phase imaging. Laser Photon. Rev..

[25] Lim J (2015). Comparative study of iterative reconstruction algorithms for missing cone problems in optical diffraction tomography. Opt. Express.

[26] Kim K, Shin S, Park Y (2016). Principles and applications of three-dimensional holographic microscopy. Polymer Sci. Technol..

[27] Park Y, Depeursinge C, Popescu G (2018). Quantitative phase imaging in biomedicine. Nature Photon.

[28] Joo C, Akkin T, Cense B, Park BH, De Boer JF (2005). Spectral-domain optical coherence phase microscopy for quantitative phase-contrast imaging. Opt. Lett..

[29] Vicar T, Raudenska M, Gumulec J, Balvan J (2020). The quantitative-phase dynamics of apoptosis and lytic cell death. Sci. Rep..

[30] Hsieh H-C, Lin P-T, Sung K-B (2022). Characterization and identification of cell death dynamics by quantitative phase imaging. J. Biomed. Opt..

[31] Lu S, Tian Y, Zhang Q, Lu X, Tian J (2022). Dynamic quantitative phase imaging based on ynet-convlstm neural network. Opt. Lasers Eng..

[32] Jo Y (2021). Label-free multiplexed microtomography of endogenous subcellular dynamics using generalizable deep learning. Nat. Cell Biol..

[33] Kang I, Goy A, Barbastathis G (2021). Dynamical machine learning volumetric reconstruction of objects’ interiors from limited angular views. Light Sci. Appl..

[34] Hore, A. & Ziou, D. Image quality metrics: Psnr vs. ssim. In *2010 20th international conference on pattern recognition*, 2366–2369 (IEEE, 2010).

[35] Kim, H. *et al.* Deep learning-based 3d refractive index generation for live blood cell. In *2022 IEEE International Conference on Bioinformatics and Biomedicine (BIBM)*, 3833–3835 (IEEE, 2022).

[36] Wang Z, Bovik AC, Sheikh HR, Simoncelli EP (2004). Image quality assessment: from error visibility to structural similarity. IEEE Trans. Image Process..

[37] He, J. *et al.* Exploring the limits of differentially private deep learning with group-wise clipping. In *The Eleventh International Conference on Learning Representations* (2023).

[38] Bu, Z., Wang, Y.-X., Zha, S. & Karypis, G. Automatic clipping: Differentially private deep learning made easier and stronger. *Adv. Neural Inf. Process. Syst.* **36** (2024).

[39] Menon, A. K., Rawat, A. S., Reddi, S. J. & Kumar, S. Can gradient clipping mitigate label noise? In *International Conference on Learning Representations* (2019).

