# Supplementary material for: Deep-learning based 3D birefringence image generation using 2D multi-view holographic images
Source: Sci Rep. 2024 Apr 30;14:9879. doi: 10.1038/s41598-024-60023-8 (PMC11059389; doi:10.1038/s41598-024-60023-8)
Supplement: Supplementary file 1 — Supplementary Legends. [file 41598_2024_60023_MOESM1_ESM.docx]

Supplementary file – Information of Supplementary videos

Supplementary video S1: The 360-degree rotation for sample 1 (Ground truth).

Supplementary video S2: The 360-degree rotation for sample 1 (Estimation).

Supplementary video S3: The 360-degree rotation for sample 2 (Ground truth).

Supplementary video S4: The 360-degree rotation for sample 2 (Estimation).

Supplementary video S5: The 360-degree rotation for sample 3 (Ground truth).

Supplementary video S6: The 360-degree rotation for sample 3 (Estimation).
